# The Foundations of Agency – and Ethics?

**DOI:** 10.1007/s11406-016-9702-2

**Published:** 2016-04-10

**Authors:** Olof Leffler

**Affiliations:** School of Philosophy, Religion and History of Science, University of Leeds, Woodhouse Lane, Leeds, LS2 9JT UK

**Keywords:** aims of action, Nietzschean constitutivism, Humean constitutivism, reasons for action, reasons internalism

## Abstract

In this article, I take off from some central issues in Paul Katsafanas’ recent book Agency and the Foundations of Ethics. I argue that Katsafanas’ alleged aims of action fail to do the work he requires them to do. First, his approach to activity or control is deeply problematic in the light of counterexamples. More importantly, the view of activity or control he needs to get his argument going is most likely false, as it requires our values to do work that they are too fickle to do. Second, I take issue with the Nietzschean drive psychology underlying the second agential aim, viz. power. I argue that ordinary desires better describe a number of phenomena that Katsafanas uses drives to explain, and that some actions can aim in the opposite direction. As only drive-motivated actions aim at power, action does not, therefore, constitutively aim at power. Finally, I sketch a Humean approach to constitutivism, and argue that it both explains the desiderata that Katsafanas posits as well as solves the problems for his view. The Humean view is preferable, and should be developed further.

Constitutivism – roughly, the view that normative requirements or standards are based on the constitutive features of action or agency^1^ – has recently gained much ground among philosophers concerned with normativity and its sources. By now, many writers have defended different versions of the view,^2^ and several core problems have been discussed at both length and depth in the literature.^3^

Paul Katsafanas has recently developed a version of constitutivism, particularly in his book *Agency and the Foundations of Ethics*: *Nietzschean Constitutivism* (2013). From here and on, I refer to it as (*AFE*). *AFE* is in many ways an excellent book, but I take issue with its main claims. Katsafanas’ case for a Nietzschean constitutivism includes arguing that action has two constitutive aims: Activity and Power. Constitutive aims generate criteria for success – in this case, successful action – which are inescapable for the agent, and therefore, constitutivists think, normative.

In section (I), I describe his view in greater depth. In section (II), I first argue that Katsafanas’ case for Activity is badly flawed. His account of Activity is false because it fails to capture a kind of luck (that I call Reparatory Luck) and is compatible with a severe kind of passivity in action. Moreover, Katsafanas cannot revise his view to solve these problems, for no view which is structurally similar to Activity is likely to be correct. This is because it relies on approval of actions in virtue of values, but values are too fickle to serve this function. Then, in section (III), I criticize Katsafanas’ claim that all actions aim at Power, and therefore the claim that Power is a constitutive aim of action. I argue, first, that because interpreting actions as motivated by desires rather than drives is less controversial and more parsimonious than adding drives, we should think purported counterexamples do not stem from drives. Second, his response to counterexamples where agents act to minimize or are indifferent to resistance is lacking.

But I also do something constructive. In section (IV), I sketch the outlines of a Humean version of constitutivism and show how it can make sense of the *desiderata* that Katsafanas wants his own view to make sense of. Moreover, it solves the problems for Katsafanas’ Nietzschean view, as presented in (II) and (III). There seems, then, to be no reason to prefer the Nietzschean version to the Humean one. At the very least, constitutivists should prefer it to the Nietzschean view. Moreover, by responding to Katsafanas’ *desiderata* as well as potentially having other support, the Humean view has some support and seems fit for further development. So I conclude in section (V).

## (I)

As mentioned, Katsafanas thinks that Activity and Power are constitutive aims of action. What does this mean? First, briefly, according to Katsafanas, Activity is equilibrium; an action is active in case the agent performing it approves of it now or would keep doing so when possessing more knowledge about its etiology. The second aim is Power, according to which actions aim at overcoming resistance. I characterize these aims in more depth below. The simplistic characterizations will do for now, as I only aim to show how they fit into Katsafanas’ view.

They serve, then, as constitutive aims. Katsafanas characterizes a constitutive aim as follows:

(*Constitutive Aim*) Let *A* be a type of attitude or event. Let *G* be a goal. *A* constitutively aims at *G* iff

(*i*) each token of *A* aims at *G*, and

(*ii*) aiming at *G* is part of what constitutes an attitude or event as a token of *A*.^4^

Here, the idea is that action – *A* – constitutively aims at Activity and Power. These aims, Katsafanas thinks, satisfy both (*i*) and (*ii*). Hence, each action aims at Activity and Power, at least to some extent, and doing so is part of constituting events as action-tokens. Here, Constitutive Aim grounds the further claim Success:

(*Success*) If *X* aims at *G*, then *G* is a standard of success for *A*.^5^

Since action has constitutive aims, *Success* is inescapable insofar as we act. And it is Activity and Power which are the standards of success for action. These generate pro tanto reasons for action, though other pro tanto reasons are provided by an agent’s values.

Furthermore, together, Activity and Power generate normative content, for other values are assessed in their light. They do so by working in tandem. By Activity, an agent can approve of his or her action (to various degrees), depending on what they know about the action. And agents approve, or disapprove, of actions in virtue of their values. By themselves, these values are to be assessed by Power, and ought to be discarded either if they conflict directly with it or might lead to conflicts with it in the future, as the other value then serves to undermine Power, either in a direct instance or in general. Indeed, in the long run, the pro tanto reasons that stem from Power win out. They weight other reasons in favour of the aims consistent with Power, because they are ubiquitous, pervasive, and are (typically) reinforced by other motives.^6^

## (II)

I take issue with Katsafanas’ aims, however. In this section, I discuss Activity, and argue that it has three main problems. Note first that when presenting his positive account, Katsafanas emphasizes that “Activity” is his own term. As he says: “I will use ‘agential activity’ as the most general term for notions that are meant to pick out the agent’s contribution to the production of action.”^7^ However, “Control” can be used instead of this term, and indeed, I shall use the terms Activity and Control interchangeably below instead of “agential activity,” for this enables me to say more about the phenomenon Katsafanas is after, i.e. the agent’s own contribution or to her action.^8^

Katsafanas is, admittedly, somewhat vague about what he is after with Activity. He lists a set of terms used by different authors – “*identification*, *wholeheartedness*, *guidance by the agent*, *direction by the agent*, *agential control*, *agential activity*, *reflective self-control*, *rational control*, and so forth”^9^ – without clarifying exactly how, if at all, they hang together. It is possible that the authors he tries to unify as discussing the same kind of thing are discussing different phenomena. However, he adds that he uses “‘agential activity’ as the most general term for notions that are meant to pick out the agent’s contribution to the production of action. Agential activity is a genus whose species are [the phenomena just mentioned].”.^10^

There seems to be *something* he is onto, however, even if not all authors he mentions are discussing the same thing.^11^ There certainly seems to be something special that we at times can contribute to an action, by steering it in the right way, to make it a paradigmatic action. “Control” is an apt word for that, because it is emphasizes that it is through the property characterized here that agents steer their actions by contributing something to them.

There are two reasons in particular to think that Katsafanas must account for a strong form of control that does not action be steered by features external to the agent. First, as just mentioned, it seems that whatever the agent’s contribution to an action is, the agent does not contribute to the success of the action if it succeeds in virtue factors that the agent does not control (in a very ordinary sense of “control”). Second, Katsafanas listed many forms of control as species of the genus which is activity, so accounting for the intuitions underlying these are *desiderata* for a full account of Activity (or, as it were, Control, in a broad sense of that word).^12^

To be fully active, then, whatever else a full account of Activity or Control contains, agents must at least control their actions both when deciding what to do and when enacting that decision, for these are different places where their contributions could be interrupted. And, among other things, paradigmatic cases of loss of this kind of control stem from various kinds of desires overwhelming the agent, or the intervention of external circumstances that makes agents lose direction. In both cases, the agent contributes less to the outcome than otherwise.

Katsafanas then goes on to deny an account of this phenomenon which he ascribes to Kant and Locke, according to which reflection on one’s motives for acting involves, at least, suspending the influence of these motives on the action one is about to perform, so that the motives can incline without necessitating the action to be performed.^13^ I will not discuss his criticism of this view further, however; instead, I will go on to criticize Katsafanas’ own positive suggestion. Expanding on the brief characterization of Activity above, Katsafanas’ account looks as follows^14^:

(*Activity*) All actions manifest some degree of *Activity*, and more *Activity* the more it is in (*Equilibrium*) and not (*Disequilibrium*), where

(*Equilibrium*) means that: “The agent *A*’s and approves of her *A*-ing. Further knowledge of the motives that figure in *A*’s etiology would not undermine her approval of *A*-ing” and

(*Disequilibrium*) means that: “The agent *A*’s and currently approves of her *A*-ing. However, if she knew more about the motives that figure in A’s etiology, she would no longer approve of her *A*-ing.”^15^

From now and on, italicized *Activity* refers to Katsafanas’ view. But when I write Activity without italicization, I use the term in general. The question, then, is how good *Activity* is as an account of Activity or Control.

There are three reasons to think that it is not very good. Two are counterexamples. Out of these, first, Activity does not seem to capture a kind of luck that I call “Reparatory Luck.” Generally, by Reparatory Luck, I mean the luck involved in situations where an agent *A*_*g*_ tries to perform an action *A* and the aim *A*_*i*_ of *A* would not have been reached by *A*_*g*_’s efforts, but *A*_*i*_ still ends up being reached due to the intervention of arbitrary external circumstances.^16^ For example, a mugger (*A*_*g*_) attempts to shoot a man in the head (*A*). He misses, but the shot ricochets and hits the man (*A*_*i*_).^17^ *Activity* should capture this kind of luck because it seems that *A*_*g*_ is inactive, or not in control of, reaching *A*_*i*_ when Reparatory Luck is present. *A*_*g*_ would have failed to reach his aim had the external world not luckily cooperated.

But what does *Activity* imply with respect to Reparatory Luck? Consider the case above. Shooing and missing, but then hitting due to a ricochet, seems compatible with keeping on approving of the action in light of further knowledge about the etiology of one’s action. For the mugger still hits his original aim, after all. Therefore, the shot was active according to *Activity*, so he can remain in *Equilibrium*. But what has happened is an instance of Reparatory Luck. And saying that such actions are active or controlled, in the sense according to which active or controlled actions are steered or authored by the agent rather than by external circumstances, certainly seems too inclusive. It is a lucky external circumstance that makes the shot ricochet.

The second problem with *Activity* is that it seems possible to remain largely passive or uncontrolled while still being in *Equilibrium*. Suppose an agent *A* is a detached observer^18^ of all internal thoughts, feelings, desires and so on. *A* can be described as being alienated from them, experiencing being in the grip of these mental states. But *A* does not experience actively engaging with feelings and thoughts; the mental activities just seem to happen inside the agent. It seems possible to do so while remaining active according to Katsafanas’ proposed view, for gaining more knowledge could also be passively achieved in this manner, and one could continue to approve of one’s action throughout the entire episode. So the detached observer would do everything right according to *Activity*.

But, intuitively, there is a kind of Activity or Control involved in engagement that this agent lacks. With different motives, *A* would have done something else instead, but not in virtue of thinking that this other thing was better, more reasonable, rational, or whatever, but just because of these different motives. If so, the agent does not seem to be active or control his actions in the sense Katsafanas attempts to capture, for here it is whichever arbitrary desires the agent has that activate or – indeed – control *A*.^19^

My diagnosis is that *Activity* is too minimal a criterion to capture all the relevant aspects Activity or Control. It includes too much. And, importantly, it would not help to just amend it by adding the anti-luck clause to solve the first problem, and an anti-passivity clause to solve the second. The fact that it suffers from both of these problems shows that it would be ad hoc. A deeper amendment is needed.

I leave it open how that amendment would look, however, for there is no obvious consensus about what is a proper view here.^20^ If it is open which one is right, however, it is unclear which it is that one should accept. So until a version is systematically defended, or at least until we can be secure that we know some features that will be part of a comprehensive account, it is unclear which normative implications Activity has. Hence, drawing important consequences from it seems far too early. So it seems implausible to think that one of the aims of action is *Activity* in Katsafanas’ sense.

Moreover, there is a third main problem with *Activity*, though it differs from the ones above insofar as it is not a problem with its content, but with its interplay with Power. Katsafanas needs a version of this aim with the same structural features as his view, according to which an action is approved of or not in virtue of deeper values (that, in turn, can be assessed by Power). For if it does not look like this, he cannot combine it with Power to generate the kind of normative claims that he defends.

But all views with this structure seem to put the cart before the horse. Approval in the light of values, in an ordinary sense of what a “value” is, can be paradigmatically uncontrolled. The values in question could be manipulated by someone else, disproportionate emphasis might be placed on some values over others, and so on and so forth. Such values are instilled by others (e.g. manipulative politicians or clergy), or by the agent’s own errors (e.g. when the agent fails to judge which matters the most), without the agent noticing the errors or, alternatively, even retaining the values by wishful thinking or similar when shown that they are wrong. Such values can hardly be said to be active or under the agent’s control, and accordingly assessments in their light cannot be controlled either.

Katsafanas might deny that values are so uncontrolled, however. On the face of it, he might then reply that the relevant values are (to be) assessed by Power. But he cannot help himself to this deeper standard. Power is not, as I shall argue in the next section, a constitutive aim of action.

But even if it were, it would not be helpful – it seems extensionally inadequate to ground an account of what it means to be active or to control an action. This is because it generates a standard of success where one succeeds if one overcomes various forms of resistance. Katsafanas argues that it could be violated directly or indirectly, so that one either aims to overcome resistance to an insignificant extent, or acts in ways (or has values) that disallow one to do so further in the future.

But then, take one of Katsafanas’ Nietzsche-inspired examples of someone who has values that lead them to violate this standard: Religious ascetics. Assuming they are successful ascetics, they certainly seem to be in great control of themselves.^21^ Katsafanas should think that they are not, however, because they are “active” in virtue of values that cannot be sustained in virtue of Power. Such values cannot be subsumed under *Activity*, however, for they fundamentally clash with Power and must therefore be rejected on reflection. So ascetics are under the spell of an ideology that cannot be rationally upheld on his view, and hence do not count as exercising *Activity*. But this is awkward. Potentially misguided control is still control, so there is no sense in which they are not active or controlling in their actions.

Or is it? The background ideology itself can be assessed by Power. There can be multiple levels of activity, one in specific actions, and one of background ideology, for example.^22^ Accordingly, Katsafanas could say that religious ascetics are not being active on some level, for they have underlying values that, by themselves, are inactive. So they do not count as active. But surely their actions can count as active or controlled in this particular case, even if not all of their motives are. It seems too strong to demand that *Activity* should have to assess motives all the way down, as it were, for that, too, seems extensionally inadequate. Very often, that seems unnecessary – little assessment seems needed if I value drinking a glass of water when I am thirsty, for example.

## (III)

I just argued that Katsafanas most likely is wrong about Activity, but I did not deny that it could be an aim of action. Katsafanas’ second agential aim – *Power* – does not seem to be such an aim, however.^23^ In this section, I try to clarify what Power is, and, somewhat unspectacularly, argue that the drive psychology which Katsafanas uses to argue that Power is an aim of action is false.

To make these points, I first need some terminology. Katsafanas does not spell out what power means in as detailed a manner as he defines *Constitutive Aim*, *Success*, *Equilibrium* or *Disequilibrium*, but he is clear that he is not talking about everyday (or social scientific) dominance-based takes on what power is. A characterization as good as any for present purposes is:

(*Power*) An agent *A* has Power (to some degree) over an object *O* if *A* intentionally overcomes a resisting object *O*, where

(*i*) “object” specifies the aim of the act of overcoming, whatever it is, that provides resistance for the completion of the action, and

(*ii*) “intentionally overcomes” means that *A* intentionally succeeds in understanding, using, or becomes able to use *O* (to some degree).

For example, *A* overcomes, and therefore has *Power* over, the aim of playing the guitar (*O*) if *A* learns how to play the guitar. And *A* can master playing the guitar to various degrees. According to Katsafanas, Nietzsche’s will to power is to be understood as the activity of overcoming resistances in this way: “[T]o will power is [to perpetually] seek to encounter and overcome resistance in the pursuit of some end.”^24^ And willing *Power* in this sense, Katsafanas thinks, is present in every token of action.^25^

Of course, such a position needs defense. The argument for saying that *Power* is an agential aim is based on a Nietzschean drive psychology. Katsafanas tries to reconstruct Nietzsche’s view, and also defend it with some an argument from the nature of satisfaction. Like Katsafanas himself, I am primarily concerned with his positive case rather than his attempt at Nietzschean exegesis (though I return to these issues in more detail towards the end of this section). The argument for the constitutive drive psychology, then, goes like this:

First, drives are motivational states that aim at their own expression, and take various objects merely as chance occasions for expressions. Second, drive-motivated actions constitutively aim at encountering and overcoming resistance. Third, all human actions are drive-motivated. It follows that all human actions inescapably aim at encountering and overcoming resistance. (…) [A]ll human action manifests will to power. Power is a constitutive aim of action.^26^

Some clarifying comments are needed before I start discussing the argument. What is a drive? They have four features, Katsafanas thinks. They are (*i*) dispositions generating affective orientations, (*ii*) for drives, one must distinguish between their aims and their objects, (*iii*) they make agents disposed to seek out their aims, not their objects (as desires would), and (*iv*) drives are constant.^27^

More specifically, (*i*) says that drives induce representations of the world to be affectively charged. For example, food looks more appealing for a hungry agent. Furthermore, (*ii*) says that they are such that their aims can be achieved by different objects. Different kinds of food can satisfy hunger. Moreover, (*iii*) drives aim not at their objects but at their own expression. To continue the food example, an agent seeks a meal (object) to eat to satisfy his hunger (aim), not the meal for its own sake when driven by hunger. And (*iv*) the drive to eat is constant, even though it can be temporarily satisfied. But it will recur, so a temporarily full agent will presumably seek out food to eat again in the future.

Drives aim at overcoming resistance because they are aim-oriented rather than object-oriented (cf. (*iii*)). Aiming at their expression then means that they are directed towards being continuously acted upon over time, not just fulfilled at a single temporal instance, in the way, Katsafanas thinks, a desire might be. It follows that actions that, in Katsafanas’ terminology, are motivated by drives are process-directed, not goal-directed (as they would have been if they were motivated by ordinary desires). Process-directed actions aim not at their own satisfaction, but at continuously succeeding at understanding, using or becoming able to use their aim. Therefore, they aim at *Power*. For example, presumably, the action of eating is a way in which the drive may reproduce itself by allowing the agent possessing it to become hungry again. And as it is part of the structure of the drive’s aim to find new objects to overcome, insofar as the agent engages in process-directed action, she aims at continuously overcoming resistance. Katsafanas thinks that all actions are process-directed like this.

Is this argument defensible? I leave the first and second premises for now. As Katsafanas is aware, one does wonder if the third premise is right, however. Do *all* actions have such a drive-like structure? Take the action of writing this paper. Is there really a drive which can take academic writing as its object? Is it not better interpreted as motivated by a desire (or whatever someone with a non-Humean psychology might posit to explain it)? Of course, this is not to say that there is nothing drive-like out there. Desires to eat, for example, are surely not generated purely by Pavlovian conditioning – at times we are, perhaps, disposed to desire what makes us maintain our functions. But that is still very different from Katsafanas-style drives; for example, such drives do not require an aim/object distinction. So why are Katsafanas’ drives needed?

One can formulate this potential problem for the third premise as a dilemma. Either all actions stem from drives, or they do not. If they do, the conclusion of the argument is vindicated; all actions aim at *Power*. But if not, then not all actions aim at *Power*. If so, according to *Constitutive Aim*, where “each token of *A* aims at *G*”, not all actions aim at overcoming resistance. So either all actions stem from drives, or *Power* is not a constitutive aim of action.

Katsafanas attempts to defend the first horn. He offers some positive arguments, but also spends some time replying to criticisms of the third premise. I focus on his attempts to ward off two kinds of counterexamples, though I return to the positive arguments towards the end. Katsafanas uses two strategies to attempt to ward off the first kind of counterexamples, such as the drive-to-academic-writing above. I argue that they both fail.

An underlying problem when he discusses both kinds of counterexamples is that drives seem to be an unnecessary theoretical construct. Many writers – including Katsafanas – admit that desires (at least sometimes) provide a motivational force that brings about actions. And writers that do not tend to admit that something else than drives do. Accordingly, adding drives to agents’ motivational structures adds an extra dimension to action explanation. It is both an uncontroversial starting point that they are not needed (in his sense), and unparsimonious to add them to one’s explanations. So if we can interpret the cases without adding drives, we are better off. So that is what I am going to try to do. Katsafanas does not see how deep this problem goes, however, and takes them on as straight counterexamples.^28^

To start off, then, Katsafanas’ strategy with respect to the first kind of counterexample is to try to show how actions that seemingly do not involve drives in fact do. And that may well be plausible, but it will be enough to show that there is no need to invoke drives to explain them to be better off. He takes on a case where an agent moves a pen across a page when writing. How do these movements aim at overcoming resistance? Katsafanas suggests that such cases can be explained by claiming that they are parts of larger actions. The agent does not move the pen across the page for the sake of doing so, but for the sake of writing a text. And writing a text, in turn, is usually instrumental to yet another ulterior end too. Or, more generally:

(*Larger*) An action, *A*-ing, is a part of a larger action, *B*-ing, iff *A*-ing and *B*-ing have a common causal history.^29^

In virtue of *Larger*, minor actions can be re-described as parts of greater ones that more plausibly might be taken to aim at resistance. Katsafanas defends it with two considerations. First, he follows Nietzsche in thinking that drives generate desires, so desires stem from drives. If desires are causally generated by drives, and *Larger* is true, then desire-directed actions are part of drive-directed ones. Second, Katsafanas invokes the aim/object-distinction from (*ii*); drives can manifest themselves in several different actions, so several actions can be causally traced back to the same drive. The causal history of an action displays its ultimate ground in a drive.

It should be uncontroversial that *Larger* – or something like it – explains an important feature of many actions insofar as they are means to achieve greater ends. However, this does not imply that drives lie behind every action. Actions might just as well be traced back to desires (or what non-Humeans think does the same work) in virtue of such a principle. Hence, the first point Katsafanas makes to defend *Larger* is unimpressive.

The second point fails too. That drives might generate many kinds of action does not imply that desires could not generate some actions. Katsafanas seems content to rely on postulating the thesis that drives generate all desires, not just some of them. This would means that all desires depend on drives in some interesting sense – presumably a causal sense, to make the argument go through without further tweaking. He does not present an argument for why all desire would do so, however. But why should we not think of some desires, and then the actions stemming from them – e.g. my desire watch Tottenham Hotspur play football – as purely dependent on ecological circumstances, plus some disposition to generate desires? Or why are they not just desires depending not on external but internal circumstances? For example, why is not a desire to contemplate the eternal ideas, stemming from the mere fact that I have the concepts of what Aristotle took to be the eternal ideas, generated just by me possessing those concepts, plus some disposition to generate desires (and, arguably, some romanticizing of philosophy)?

To be sure, it is *possible* to come up with some way that these desires or actions depend on drives, just as it is often possible to interpret the same empirical findings in many theoretical frameworks in other cases. But in the light of the reasons to favour desire-based explanations (or similar) to drive-based ones above, and absent an argument for Katsafanas’ thesis that all desires depend on drives, it seems strained to force desires into his framework. So there is no reason to believe that all actions are so large that they need to be squeezed into a drive-oriented framework by *Larger*.

Katsafanas attempts to discuss a second kind of cases as well. Here, agents do not seem to aim at resistance, either because resistance plays no role in these cases, or because agents seem to try to minimize it. He tries to re-describe them as cases where the agents do aim at some minimal kind of resistance. And then he does the same thing for cases where agents in fact seem to aim to minimize resistance. His examples are a casual chat with a friend or watching a sitcom on TV. And surely, it is indeed rare that one attempts to watch a sitcom while maximizing the contrast on the screen to make it harder to look at.

Again, Katsafanas presents two possible replies to what happens in these cases. First, he argues that the resistance sought is related to the action. For example, in a casual conversation, there is simply little resistance to overcome. Second, even when there appears to be little in the way of resistance around, there is some. For example, attending to the TV show offers at least one kind of minimal resistance.

The first reply seems alright so far as it goes. Some actions – including those in the examples – clearly seem to involve very little in the way of resistance, in contrast with actions constituting more difficult ventures, such as writing a symphony or completing grad school.

However, this explanation does not go very far. In reply to both of the replies, one might ask if the best explanation of what the agents do is to seek minimal resistance, or if such cases really show that it is best to explain the kinds of resistance faced as just being in the way of completing the action. On the face of it, it seems contrived to claim that actions where agents encounter little resistance in fact aim at that resistance. Rather, when having a casual chat with a friend or (especially) when watching TV, any kind of resistance seems to be at best an annoying distraction. The aim seems simply to say what one has to say, or to be engulfed in the story.

This reinterpretation of the cases is reinforced by a second point. There are obviously various kinds of resistance present for various forces in non-action events as well. A shelf holds a book above the floor. All kinds of forces are in the way of all kinds of events. So there is no obvious reason for saying that Katsafanas’ resistance in fact is part of the structure of such actions, rather than just being in the way. So the second strategy seems implausible too.

Accordingly, neither defensive strategy work to defend Katsafanas’ claim that all actions aim at resistance. There is, however, a general objection against my criticism of Katsafanas’ defense of the first horn in the dilemma. This objection is that I might have been unfair by discussing potential implications of his view rather than the arguments in its favour. For if there is independent support for thinking that all actions stem from drives, the putative counter-examples might be uninteresting. It is still an established theory.^30^

I do not think the view has enough support to warrant this judgement, however. Katsafanas presents two styles of argument in favour of his main conclusion. The first kind of arguments is quasi-exegetical, insofar as the arguments reach back to Nietzsche’s own arguments in favour of the view that action does not aim at achieving specific aims but at continuous satisfaction. It is not clear if Katsafanas fully endorses rather than just mentions the Nietzschean lines of thought. Nevertheless, he mentions the idea that seemingly non-drive-guided actions can be reinterpreted as drive-guided actions, and the thought that some of our most lastingly satisfying actions seem to stem from drives insofar as they aim to overcome resistance. Here Katsafanas’ example is creativity: With Nietzsche, he thinks that creative individuals do not aim at producing works of art, but at the process of creative activity.^31^

I run these two lines of thought together in a way that Katsafanas does not, however, for the reason that they seem to succumb to the same counterargument – the fact that these phenomena *can* be interpreted as stemming from drives is no reason to believe that the interpretation are apt. All phenomena can be described in many ways, and we need reason to believe that some descriptions are better than others. For that we need independent arguments. If there are arguments worth pursuing in Nietzsche’s writings on these issues, Katsafanas has not presented them in *AFE*.

More interestingly, secondly, Katsafanas appeals to some empirical evidence about the nature of satisfaction to support the view that we act based on drives rather than desires. There is some evidence, he thinks, for the view that “human beings are most satisfied when engaged in activities that provide them with challenges that are neither too easy nor beyond their capacities.”^32^ And that is supposed to support the view that engaging in some processes (i.e. by process-directed actions) provides lasting satisfaction at least during the time one engages in them, as opposed to achieving ends (i.e. by goal-directed actions), which he thinks never leads to lasting satisfaction. Therefore, the evidence also supports the view that actions aim at *Power*.

However, first, even Katsafanas just seems to take the findings to be evidence for the weaker claim that there are *some* processes that provide satisfaction, not that all actions work in this way. Hence, he has no argument against the view that some (other) actions do not lead to lasting satisfaction. Moreover, second, it is unclear if this empirical point cannot be handled by saying that the processes do not aim at reaching lasting satisfaction, but rather the satisfaction of some hard-to-satisfy desires. So Katsafanas’ positive case for drives does not establish that all actions stem from them; in fact, it is still not clear that he has established that any actions stem from drives.^33^

Summing up, I have argued against the *Larger*-based case for interpreting actions as drive-based that it seems compatible with treating desire-satisfaction as the aim of (some) actions. And I have argued against Katsafanas’ second batch of counterexamples, according to which one (often) seeks to minimize resistance, that where he thinks there are few kinds or minimal degrees of resistance, these are better interpreted as just being in the way of the aim of the action than aimed at. Desires which are not generated by drives seem to be at work in explaining at least some actions, and at times actions do not seem to aim at resistance and its overcoming but to minimize it. Hence, not all actions appear to be motivated by drives. So *Power* is not a constitutive aim of action.

## (IV)

My case so far has been negative. But in this section, I aim to spell out how a Humean approach to constitutivism can look. I also explain how it can respond to all of Katsafanas’ *desiderata* for what a good form of constitutivism should do as well as solve the problems for his theory presented in the last two sections. Accordingly, if one is impressed with Katsafanas’ arguments, then one should prefer the Humean alternative. However, this does not mean that I take the Humean alternative as I present it to be the best theory all-things-considered just in virtue of the arguments presented here. Instead, I mainly aim to show that it is better than at least Katsafanas’ view, and because it has some support in virtue of its problem-solving abilities, constitutivists ought to take it seriously and develop it fully. Doing so, however, is a task for another occasion.

How does the view look? There seems to be a number of views in the literature going under the same name. I do not mean to be speaking about the kind of Humean constitutivism – associated with Jamie Dreier – that Katsafanas discusses early in *AFE*. This view just says that a minimal norm of instrumental rationality is partially constitutive of action. Nor will I discuss the constructivist view proposed by Sharon Street that Katsafanas calls a Humean version of constitutivism.^34^ On this view, agents evaluate their reasons in terms of their other reasons from a distinctly practical point of view.

Rather, the kind of Humean constitutivism I have in mind is more closely related to, though not identical with, the kind recently defended by Michael Smith. It has three main features. First, it assumes at least a (broadly) Humean belief-desire framework for explaining the constitution of action, where the presence of a belief and a desire, suitably related, is a necessary condition for construing an event as an action. They are just necessary, however; other potential features of action explanation are intentions and, importantly, rational principles – or dispositions – linking the previously mentioned states together.

Moreover, two other aspects of Smith’s 1990s apparatus also feature in this account.^35^ These are, first, the view that reasons depend on what an ideally rational counterpart would do (or desire), giving us a kind of reasons internalism, and, second, rationalist internalism about normative motivation, according to which, roughly, an agent gains a desire to φ if she judges that she ought to φ and she is rational. One needs not commit oneself to the details of Smith’s view here, however. For example, it does not really matter if the ideal counterpart is an exemplar or an advisor,^36^ or whether or not rationalist internalism is a conceptual or metaphysical claim.

The kind of reasons internalism involved in this constitutivist picture is importantly qualified, however. For one can argue, generalizing from Smith’s recent arguments,^37^ that it is somehow constitutive of the ideally rational counterpart to have certain specific, dominant desires. A dominant desire is a desire that dominates all others of the ideally rational agent. If these desires have content which is even remotely moral, and the dominant desires of an ideally rational agent (whichever they are) constitute our dominant reasons, then we suddenly seem to have dominant reasons which also are moral reasons.

One version of this strategy has been proposed by Smith. He has argued that, to remain ideal or ideally rational (I use the term synonymously below), when using her rational capacities, an ideally rational agent needs a certain set of desires to aid and not interfere with her present rational deliberation that can make her rely on herself as well as be vigilant to the risks involved in losing control. These desires also dominate all other desires, and, importantly, they generalize, Smith argues, to aiding in the exercise of, and not interfering with, others’ rational abilities.^38^ Adding his reasons internalism to this view, the ideal agent’s desires become our reasons.

I personally prefer another version of this view where the dominant desires of the ideal agent are directly rationally required because they are required by some requirement of rationality. Assuming the ideal agent makes this judgement – which surely she does, *ex hypothesi* – then by rationalist internalism about motivation, she also attains the desire. And then, by reasons internalism, we attain the reason. Whether or not there is such a requirement of rationality is another question, however, but the framework is just another instance of the general strategy, so which version is the best does not matter for present purposes.

It does matter, however, if these views are interpreted as coherentist or foundationalist constitutivisms, where the former views spell out the content of rationality (that constitutes the ideal agents) and morality at the same time in reflective equilibrium, and the latter try argue for moral principles from some general principles that are independent of moral intuitions in reflective equilibrium. Smith’s view has been associated with the coherentist approach,^39^ but in fact his argument can be read foundationalistically. I propose that this is a stronger interpretation of the view (whether or not it is Smith’s intended one), in part because it does not expose the view to ordinary problems associated with reflective equilibrium, and in part because it can help solving one of Katsafanas’ problems (as I shall argue).

Here, Katsafanas’ view is supposed to make sense of several *desiderata* for a plausible explanation of practical normativity. These are also classic problems for many traditional metaethical theories, so the fact that it can make sense of them is an argument in its favour in general. First, there is an epistemic challenge. Katsafanas’ version is an argument from disagreement. Moral disagreement over time and space, he thinks, is best explained by the preponderance of non-rational (sociological and psychological) forces which do not track the truth. Therefore, we need justification for retaining our moral beliefs. Second, there is a metaphysical challenge. Values are queer, as Mackie famously argued, and, moreover, moral theories often rely on implausible assumptions about human nature. And third, there is a practical challenge. Why does morality have a motivational grip on us? Katsafanas thinks any systematic metaethical (or, broader, metanormative) view ought to face these challenges.

He also adds two other points that his own view is supposed to make sense of. These stem from his discussion of previous constitutivist proposals from David Velleman and Christine Korsgaard. Allegedly *contra* Velleman, (*a*) any plausible constitutivist view must depend on a constitutive feature which can be realized to different extents, which Velleman’s does not. And *contra* Korsgaard, (*b*) a constitutivist view must be able to rank the weight of different reasons in virtue of the normativity-constituting features of their account, but she is only able to call actions legitimate or not, depending on whether or not they are acceptable by the tests supplied by the categorical imperative.^40^ So she is unable to rank the strengths of all those maxims that pass the tests. Accounting for these *desiderata* is the core of Katsafanas’ case for a Nietzschean version of constitutivism.^41^ Hence, if the Humean view can account for them as well as solve the problems that his own view faces, it is better off than his view.

Starting with his three classic sceptical arguments, Katsafanas himself notes that a Humean metaethics presumably can deal with the metaphysical and practical challenges.^42^ With respect to the metaphysical challenge of queer properties, the Humean view is naturalistic enough, although it needs not take any stance on exactly which metaphysics are needed. But no non-natural properties (or relations) are needed either way according to Humeans. Moreover, with respect to the practical challenge, viz. the challenge of explaining how normative judgements can get a grip on agents, rationalist internalism guarantees that an agent has the desires of the ideally rational agent if she is rational herself. But rationalist internalism is part of the view that I proposed. So that problem, too, is solved.

However, Katsafanas is more doubtful about the way in which the Humean view can solve the epistemic challenge, viz. how it can escape running into the problem of explaining how non-rational factors have explained our disagreeing judgements over time and space. Here, the foundationalist structure of the proposed Humean view can solve the problem. Arguably, a coherentist form of Humeanism might not be able to do this job very well – it might end up inheriting the ordinary problems with, e.g., biased intuitions and beliefs in reflective equilibrium, or even require relativism if all potential equilibria are supposed to be equally acceptable. But if a foundationalist argument that does not rely on moral intuitions is given, then what does the epistemic challenge really amount to? An argument for moral reasons can be provided without relying on the moral views that Katsafanas thinks there is problematic disagreement about. That is, for example, what Smith seems to try to do (on one reading of his views), but there might be other arguments than his available too. Of course, this reply to the epistemic challenge is only as strong as the foundationalist arguments. If they fail, then there is no Humean argument here. But that is not problem insofar as they are successful, so there is a strategy to solve the epistemic problem.

What of the other *desiderata*, then? Katsafanas’ new *desiderata*, and the problems for his own account? The *desiderata* were (*a*) that the constitutive feature must be possible to realize to different extents, and (*b*) that a proper constitutivist view must show how one can rank the weights of different reasons. A Humean can do both. The response to (*a*) is obvious: An agent can obviously be more or less ideal in many different ways. Here, the most important is probably the way in which she may or may not fail to grasp the reasons stemming from the ideal agent to different extents. So actions can be reason-guided to different extents. And the response to (*b*) is that as the desires of an ideally rational agent are our reasons, the strength of different desires gives rise to reasons of differing strengths.^43^ And these can be ranked.

Finally, this view can solve the problems for Katsafanas’ view. Regarding the denial of *Activity* as a good account of Activity in section (II), Humeans need not rely on controversial assumptions about activity or control at all. Hence, the problems for Katsafanas-style views do not matter here. And regarding the problems for *Power* in section (III), the Humean theory of action of course does not need to rely on drives, or even theses like *Larger*, as they famously rely on beliefs and desires when explaining action. Therefore, summarizing, the Humean view can make sense of all the points that the Nietzschean one was supposed to make sense of, and is unaffected by my challenges to Katsafanas’ view.

## (V)

In section (II), I argued that Katsafanas is wrong about the nature of Activity. *Activity* seems obviously false as it cannot capture Reparatory Luck and is compatible with a kind of passivity, and assessment of action in terms of values does not seem to be the right way to generate actions. In section (III), I argued that *Power* not is a constitutive aim of action. Not all actions are drive-guided, and the best explanations of the putative counterexamples to Katsafanas’ view stand in tension with his view. Finally, in section (IV), I sketched a Humean alternative to Katsafanas’ view. I argued that it can solve the problems for Katsafanas’ view, as well as respond to his *desiderata* more broadly.

I think this shows that some version of the Humean view is better than Katsafanas’, and indeed can make sense of Katsafanas’ original *desiderata*, which may well be interpreted as an argument in its favour by itself. This says does not say that it is ultimately correct. But it ought to be taken seriously and be investigated further. However, doing so is beyond the scope of this paper.^44^

## Footnotes

1 Some may prefer to formulate constitutivism by saying that reasons stem from these features, rather than “requirements” or “standards.” I prefer the broader formulation, for not everything normative must depend on reasons.

2 Authors include Korsgaard (1996, 2009); Velleman (2009); Walden (2012); Katsafanas (2013*a*), Smith (2009, 2012); Dreier (1997), and so on.

3 E.g. the agency-schmagency problem – see Enoch (2006, 2011); Velleman, *op. cit*., Ferrero (2009), Katsafanas, *op. cit*., and Silverstein (2015), and the problem of bad action – see Lavin (2004).

4 See *AFE*, p. 39.

5 See *ibid*. However, Katsafanas qualifies this view, and claims that his version of constitutivism only requires that if an agent aims at some goal, and endorses this aim, achieving that goal is a standard of success for the action (pp. 208–209).

6 See *ibid*., pp. 39; 191–200.

7 See *ibid*., p. 113.

8 See *ibid*.

9 See *ibid*. His italics.

10 See *ibid*., pp. 113–114. My brackets.

11 See *ibid.*, pp. 111–115.

12 Many authors (e.g. Fischer and Ravizza (1998)) have tried to work out the sense of control needed to be responsible for actions. But it is a substantive question if this kind of control is the same kind of control that is often (implicitly) taken to be partially constitutive of action. Perhaps it is sub-notion of the more general notion. Moreover, control in general does not seem to be the same thing as self-control (cf. Henden 2008). Others have noticed the same gap in the literature on control-in-general, however, and accounts are emerging: cf. e.g. Shepherd (2014). I thank an anonymous referee for this journal for forcing me to be clearer here.

13 “Motives” is Katsafanas’ term. The reader is free to substitute the term for drives, desires, besires, beliefs, or other motivational states.

14 In virtue of the lack of iffs, however, I refrain from reading this as a biconditional definition.

15 See *AFE*, p. 138 for the citations.

16 Different ways of spelling out these circumstances may well yield different kinds of Reparatory Luck. I ignore this complication in the article.

17 Obviously, this kind of luck lies behind many standard cases of deviant causation.

18 Cf. Strawson (1986) for a similar character in the context of discussions about free will.

19 So, strictly speaking, it may even be wrong to call this person an ”agent” in a technical sense of the word.

20 Is this unfair to a writer such as Shepherd, *op. cit*., who has recently presented an account in terms of reliable causation over counterfactual situation? His account is not unproblematic either. For example, it suffers from the problem of passivity that Katsafanas also suffers from.

21 See *AFE*, p. 195.

22 I thank an anonymous reviewer for this suggestion.

23 Various reviewers of *AFE* has noticed that this view of Katsafanas’ seems problematic; see Ferrero (2015) and Poellner (2015). But none have developed the argument at length like I do.

24 See ibid., p. 159.

25 I follow Katsafanas in leaving the concept of “willing” unspecified.

26
*AFE*, p. 165.

27 Katsafanas (2015). Moreover, the affective orientations can also be understood as evaluative.

28 I thank an anonymous referee for making me develop this point.

29
*AFE*, p. 180.

30 An anonymous reviewer has pressed this reply against my take on the second batch of counterexamples, arguing that Katsafanas has already established his view, and therefore only needs to show that the cases he discusses are *compatible* with it, not best explained by it. But I now argue that Katsafanas has failed to establish that his view is strong enough for this strategy.

31
*AFE*, p. 171–174.

32 See ibid., p. 174.

33 See Ferrero (2015) for similar considerations.

34 See e.g. Street (2012), discussed in Katsfaanas (fc.).

35 For example, Scanlon (2014), ch. 1, criticizes such takes on reasons, but Tubert (2015) defends something like it.

36 For discussion, see e.g. Smith (2004 [1997]) and Tubert, *op. cit*.

37 Smith (2011, 2012).

38 I cannot go deeper into the argument here, however, but see Leffler (2014) for discussion.

39 By Smith (2010), and also Katsafanas (2016).

40 See *AFE*., ch. 3 & 4; p. 109.

41 At least in Katsafanas (2013). It may be that he takes on other motivations later – cf. Katsafanas (2016). But the most important arguments there are also addressed here.

42 See *AFE*, pp. 32–35.

43 Notably, however, apart from the dominant ones, these may be agent-relative. But that is irrelevant here.

44 I want to thank Caj Strandberg, Ulrike Heuer, an anonymous referee for this journal, as well as a number of my former grad student colleagues at the University of Gothenburg who have listened to a presentation of a previous version of this paper.

## References

[CR1] Dreier J, Cullity G, Gaut B (1997). Humean doubts about the practical justification of morality. Ethics and practical reason.

[CR2] Enoch D (2006). Agency, shmagency: why normativity won’t come from what is constitutive of action. Philosophical Review.

[CR3] Enoch D, Brady M (2011). Shmagency revisited. New waves in metaethics.

[CR4] Ferrero L, Shafer-Landau R (2009). Constitutivism and the schmagency challenge. Oxford studies in metaethics.

[CR5] Ferrero L (2015). Katsafanas, Paul. agency and the foundations of ethics: nietzschean constitutivism. Oxford: Oxford University Press, 2013. Pp. 267. $75.00 cloth. Ethics.

[CR6] Fischer JM, Ravizza M (1998). Responsibility and control: a theory of moral responsibility.

[CR7] Henden E (2008). What is self-control?. Philosophical Psychology.

[CR8] Katsafanas P (2013). Agency and the foundations of ethics: Nietzschean constitutivism.

[CR9] Katsafanas, P. (2015). Value, affect, and drive. In P. Kail & M. Dries (Eds.), *Nietzsche on mind and nature* (pp. 163–188). Oxford: Oxford University Press.

[CR10] Katsafanas, P. (2016). Constitutivism about practical reasons. In D. Star (Ed.), *Oxford handbook of reasons and normativity* (in press). Oxford: Oxford University Press. http://philpapers.org/archive/KATCAP.pdf. Accessed 28 Oct 2014.

[CR11] Korsgaard CM (1996). The sources of normativity.

[CR12] Korsgaard CM (2009). Self-constitution: agency, identity and integrity.

[CR13] Lavin D (2004). Practical reason and the possibility of error. Ethics.

[CR14] Leffler, O. (2014). *Rationalism with a Humean face. Do the desires of fully rational agents constitute universal normative reasons?* (MA dissertation). University of Gothenburg, Gothenburg.

[CR15] Poellner P (2015). On nietzschean constitutivism. European Journal of Philosophy.

[CR16] Scanlon TM (2014). Being realistic about reasons.

[CR17] Shepherd J (2014). The contours of control. Philosophical Studies.

[CR18] Silverstein M (2015). The shmagency question. Philosophical Studies.

[CR19] Smith, Michael (2004 [1997]). *In defence of The Moral Problem: A reply to brink, copp, and Sayre-McCord*, reprinted in Smith, Michael (2004), Ethics and the A Priori: Selected Essays on Moral Psychology and Metaethics, Cambridge: Cambridge University Press, pp. 259–296.

[CR20] Smith M, Sobel D, Wall S (2009). The explanatory role of being rational. Reasons for actions.

[CR21] Smith M, Richard J, Kirchin S (2010). Beyond the error theory. A world without values: essays on John Mackie’s error theory.

[CR22] Smith M (2011). Deontological moral obligations and non-welfarist agent-relative values. Ratio.

[CR23] Smith M (2012). Agents and patients: Or, what We learn about reasons for action by reflecting on our choices in process-of-thought cases. Proceedings of the Aristotelian Society.

[CR24] Strawson G (1986). Freedom and belief.

[CR25] Street S, Lenman J, Shemmer Y (2012). Coming to terms with contingency: humean constructivism about practical reason. Constructivism in practical philosophy.

[CR26] Tubert A (2015). Sound advice and internal reasons. Pacific Philosophical Quarterly.

[CR27] Velleman DJ (2009). How we get along.

[CR28] Walden, Kenneth (2012). Laws of nature, Laws of freedom, and the social construction of normativity. In: Shafer-Landau, Russ (ed.). *Oxford studies in metaethics*, Vol. 7, pp. 37–79.

